# Involvement of orexin type-2 receptors in genetic absence epilepsy rats

**DOI:** 10.3389/fneur.2023.1282494

**Published:** 2023-11-30

**Authors:** Aylin Toplu, Nursima Mutlu, Elif Tuğçe Erdeve, Özge Sariyildiz, Musa Çelik, Devrim Öz-Arslan, Özlem Akman, Zoltan Molnár, Nihan Çarçak, Filiz Onat

**Affiliations:** ^1^Department of Medical Pharmacology, School of Medicine, Marmara University, Istanbul, Türkiye; ^2^Department of Neuroscience, Health Sciences Institute, Acibadem Mehmet Ali Aydinlar University, Istanbul, Türkiye; ^3^Department of Molecular Biotechnology and Genetics, Institute of Science, Istanbul University, Istanbul, Türkiye; ^4^Department of Pharmacology, Health Sciences Institute, Istanbul University, Istanbul, Türkiye; ^5^Department of Biophysics, Health Sciences Institute, Acibadem Mehmet Ali Aydinlar University, Istanbul, Türkiye; ^6^Department of Biophysics, School of Medicine, Acibadem Mehmet Ali Aydinlar University, Istanbul, Türkiye; ^7^Department of Physiology, Faculty of Medicine, Demiroglu Bilim University, Istanbul, Türkiye; ^8^Department of Physiology, Anatomy and Genetics, University of Oxford, Oxford, United Kingdom; ^9^Department of Pharmacology, Faculty of Pharmacy, Istanbul University, Istanbul, Türkiye; ^10^Department of Medical Pharmacology, School of Medicine, Acibadem Mehmet Ali Aydinlar University, Istanbul, Türkiye

**Keywords:** absence epilepsy, orexin type-2 receptor, spike-and-wave discharge, YNT-185, epilepsy

## Abstract

**Introduction:**

Orexin is a neuropeptide neurotransmitter that regulates the sleep/wake cycle produced by the lateral hypothalamus neurons. Recent studies have shown the involvement of orexin system in epilepsy. Limited data is available about the possible role of orexins in the pathophysiology of absence seizures. This study aims to understand the role of orexinergic signaling through the orexin-type 2 receptor (OX2R) in the pathophysiology of absence epilepsy. The pharmacological effect of a selective OX2R agonist, YNT-185 on spike-and-wave-discharges (SWDs) and the OX2R receptor protein levels in the cortex and thalamus in adult GAERS were investigated.

**Methods:**

The effect of intracerebroventricular (ICV) (100, 300, and 600 nmol/10 μL), intrathalamic (30 and 40 nmol/500 nL), and intracortical (40 nmol/500 nL) microinjections of YNT-185 on the duration and number of spontaneous SWDs were evaluated in adult GAERS. The percentage of slow-wave sleep (SWS) and spectral characteristics of background EEG were analyzed after the ICV application of 600 nmol YNT-185. The level of OX2R expression in the somatosensory cortex and projecting thalamic nuclei of adult GAERS were examined by Western blot and compared with the non-epileptic Wistar rats.

**Results:**

We showed that ICV administration of YNT-185 suppressed the cumulative duration of SWDs in GAERS compared to the saline-administered control group (*p* < 0.05). However, intrathalamic and intracortical microinjections of YNT-185 did not show a significant effect on SWDs. ICV microinjections of YNT-185 affect sleep states by increasing the percentage of SWS and showed a significant treatment effect on the 1–4 Hz delta frequency band power during the 1–2 h post-injection period where YNT-185 significantly decreased the SWDs. OXR2 protein levels were significantly reduced in the cortex and thalamus of GAERS when compared to Wistar rats.

**Conclusion:**

This study investigated the efficacy of YNT-185 for the first time on absence epilepsy in GAERS and revealed a suppressive effect of OX2R agonist on SWDs as evidenced by the significantly reduced expression of OX2R in the cortex and thalamus. YNT-185 effect on SWDs could be attributed to its regulation of wake/sleep states. The results constitute a step toward understanding the effectiveness of orexin neuropeptides on absence seizures in GAERS and might be targeted by therapeutic intervention for absence epilepsy.

## Introduction

Orexin-A and -B (hypocretins) which are the neuropeptides mainly derived from orexin-containing neurons in the hypothalamus play a crucial role in circadian rhythm and specifically mediating wakefulness in the sleep–wake system (1–4). Orexinergic neurons project to the cortex and virtually all subcortical arousal systems and exert their effects by binding to orexin type-1 (OX1R) and orexin type-2 receptors (OX2R) (2, 5). The orexinergic system has recently been investigated in the sleep-arousal network and disorders related to the sleep architecture, such as epilepsy (6, 7). Limited evidence is available about the possible role of the orexinergic system in the pathophysiology of generalized epilepsies (8, 9). Therefore, the orexinergic system has become the focus of much attention for its possible involvement in the modulation of genetic generalized epilepsies. It has also been stated that convulsive seizures can be reduced by improving sleep quality by using orexin antagonists (10). Absence epilepsy occupies a prominent position in genetic generalized epilepsies since absence seizures are quite common, accounting for 10–17% of all cases of epilepsy diagnosed in children (11, 12). Spontaneous spike-and-wave discharges (SWDs), a pathognomonic feature of the EEG in typical absence seizures of absence epilepsy, start and end abruptly on a normal EEG background in a state of quiet wakefulness (13, 14). It has recently been shown that OX1R protein levels are significantly lower in the thalamus and the somatosensory cortex of adult genetic absence epilepsy rats from Rijswijk (WAG/Rij model) that is well-validated and commonly used genetic model of absence epilepsy (15).

Despite this initial work, the link between orexinergic signaling and absence epilepsy remains to be demonstrated. In order to better understand the link between orexinergic system and absence seizures, further studies involving other models of absence epilepsy are required. The other genetic model including naturally occurring mutations in rats from Strasbourg (GAERS), serves as a model for the approach to experimental and translational paths to the understanding of absence seizures and absence epileptogenesis (16). Revealing the role of the orexinergic system in GAERS will help solidify the knowledge in the field of absence epilepsy. In addition, the function of OX2R in the epileptogenesis of absence seizures and the *in-vivo* effects of orexin ligands on absence seizures in genetic absence epilepsy models are still unknown. Clarification of these issues will further contribute to a better understanding of the pathophysiological mechanisms of generalized genetic epilepsies related to sleep–wakefulness states.

Here we investigated whether orexinergic transmission through OX2R modulates absence seizures in rats with absence epilepsy namely GAERS. First, we demonstrated the pharmacological effect of the OX2R agonist, YNT-185, applied by intracerebroventricular (ICV) or intraparenchymal microinjections in adult GAERS. We also examined the effect of YNT-185 on slow-wave sleep (SWS) and conducted spectral analysis of EEG. Finally, we investigated the OX2R receptor protein levels by immunoblotting in the cortex and thalamus of adult GAERS and compared them with adult non-epileptic Wistar rats.

## Materials and methods

### Animals

Experiments were performed in adult (3–4 months old), 300–350 g male GAERS and Wistar rats. The experiments were performed in Acibadem Mehmet Ali Aydinlar University, *Laboratory Animal* Application and *Research Center* (ACU-DEHAM). Animals were kept within cycles of 12 h in light, 12 h in dark environment, and with a room temperature of 22–24°C. This study was approved by the Acibadem University Ethical Committee for Experimental Animals (ACU-HADYEK) conforming with the EU Directive 2010/63/EU for animal experiments (HDK-2022/36).

### Stereotaxic surgery

Animals were anesthetized using inhalation isoflurane (2.5–3%, the flow rate of oxygen was ˜0.8 L/min) anesthesia and placed into a stereotaxic instrument (Stoelting Model 51,600, Stoelting Co. Illinois, USA). For ICV microinjections, a stainless-steel guide cannulla (C313G; Plastics One, Roanoke, VA, USA) was implanted unilaterally according to the coordinates from the atlas of Paxinos and Watson (17) (AP: −1.0 mm, ML: −1.4 mm, DV: 4.1 mm from bregma). For intraparenchymal microinjections, stainless steel guide cannulas (C315G; Plastics One, Roanoke, VA, USA) were implanted bilaterally targeting ventrobasal complex of thalamus (VB) or somatosensorial cortex (S1) according to the coordinates from the atlas of Paxinos and Watson (17) (VB coordinates: AP: −3.2 mm, ML: ±4.8 mm, DV: 5.5 with an angle of 16°; S1 coordinates: AP: −2.1 mm, ML: ±5.5 mm, DV: 3.5 mm from bregma). EEG recording electrodes were implanted bilaterally over the fronto-parietal cortices. Dental acrylic was used to protect each implant onto the skull. A stainless-steel dummy was also inserted into the guide cannulas until the microinjection. Following the surgery, the animals were returned to their cages for routine care as single animals per cage, and allowed to recover for one week before the EEG recordings. The experimental design is presented in Supplementary Figure S1, and was created by using BioRender.com.

### Intracerebral microinjections

After a 1-week recovery period, each animal was placed in a plexiglass recording chamber and habituated for 40 min. Then a 3-h baseline EEG (from 9 a.m. to 12 p.m.) was recorded from GAERS in order to confirm the occurrence of typical absence seizures after stereotaxic surgery. The next day, an internal cannula for ICV or intraparenchymal (VB or S1) was inserted into the guide cannula, extending 1 mm below the tip of the guide. Then the different doses of YNT-185 solution or an equal volume of sterile saline (%0.9 NaCl) were delivered via a microinfusion pump (at a rate of 10 μL/5 min for ICV or at a rate of 500 nL/5 min for intraparenchymal microinjections). EEG was recorded for 180 min after the microinjections. The cumulative seizure duration, number of seizures, and duration of individual seizures were analyzed and compared among the groups.

### EEG recording and analysis

EEG was amplified through a BioAmp ML 136 amplifier, with band pass filter settings at 1–40 Hz, recorded and analyzed using Chart v.8.1 program (PowerLab8S ADI Instruments, Oxfordshire, UK). The total time spent in seizure, and the number and duration of individual seizure were evaluated. In both the baseline recording and the recording after administration, only SWD complexes with a train of SWD (7–11 Hz) and an amplitude at least twice that of the background EEG were found at periods longer than 1 s and were assessed (See Supplementary Figure S2). The cumulative seizure duration, the number of seizures, and the duration of individual seizures (duration of each SWD) were evaluated in the EEG recording taken over 3 h.

### Slow-wave sleep scoring and spectral analysis of background EEG

EEG sleep scoring and spectral analysis were conducted in a blinded manner by a neurophysiologist. EEG segments showing slow frequencies in the EEG background, including high-amplitude delta waves, were categorized as slow-wave sleep (SWS) following previously established criteria (18). Due to the absence of video and electromyographic recordings, it was not possible to score active wakefulness, passive wakefulness, and REM states. The percentage of SWS was quantified in both the 600 nmol/10 μL ICV YNT-185 group, in which YNT-185 significantly suppressed SWDs, and the saline-treated vehicle group. The 3-h EEG recordings following YNT-185 or saline administration were compared to baseline EEG EEG recordings obtained one day before the injections in each group. To confirm the SWS scores obtained from EEG recordings of the 600 nmol/10 μL-YNT-185 group, in which YNT-185 significantly increased SWS, and to evaluate other frequency changes due to YNT-185 administration, the spectral characteristics of the background EEG were analyzed by computing the power spectra using fast Fourier transform (FFT; MATLAB 9.5, MathWorks), as previously described (19). To assess the effects of YNT-185 on EEG characteristics, we analyzed 0–1 h, 1–2 h, and 2–3 h post-injection EEG data from the right cerebral cortex of animals and compared it to its baseline EEG recordings. FFT was performed on 2000 randomly picked artifact-free epochs of 2-s duration of background EEG, without any SWD pattern, tapered with a Hamming window, and a frequency resolution of 0.5 Hz. The absolute power spectra were divided into 1–4 Hz (delta), 4.5–8 Hz (theta), 8.5–13 Hz (alpha), 13.5–30 Hz (beta) frequency bands, and the mean power for each of these frequency bands was computed.

### Histological verification

The location of each cannula was confirmed by histological verification. After the EEG recordings, the location of the ICV cannula was confirmed by anesthetizing each rat, inserting a needle into the guide cannula, and injecting 10 μL of 1% methylene blue into the lateral ventricle. The needle was kept at the injection site for at least 60 s before removal. After decapitation, the brain was removed, and the traces of methylene blue were inspected to determine the sites of ICV injection.

**Figure 1:**
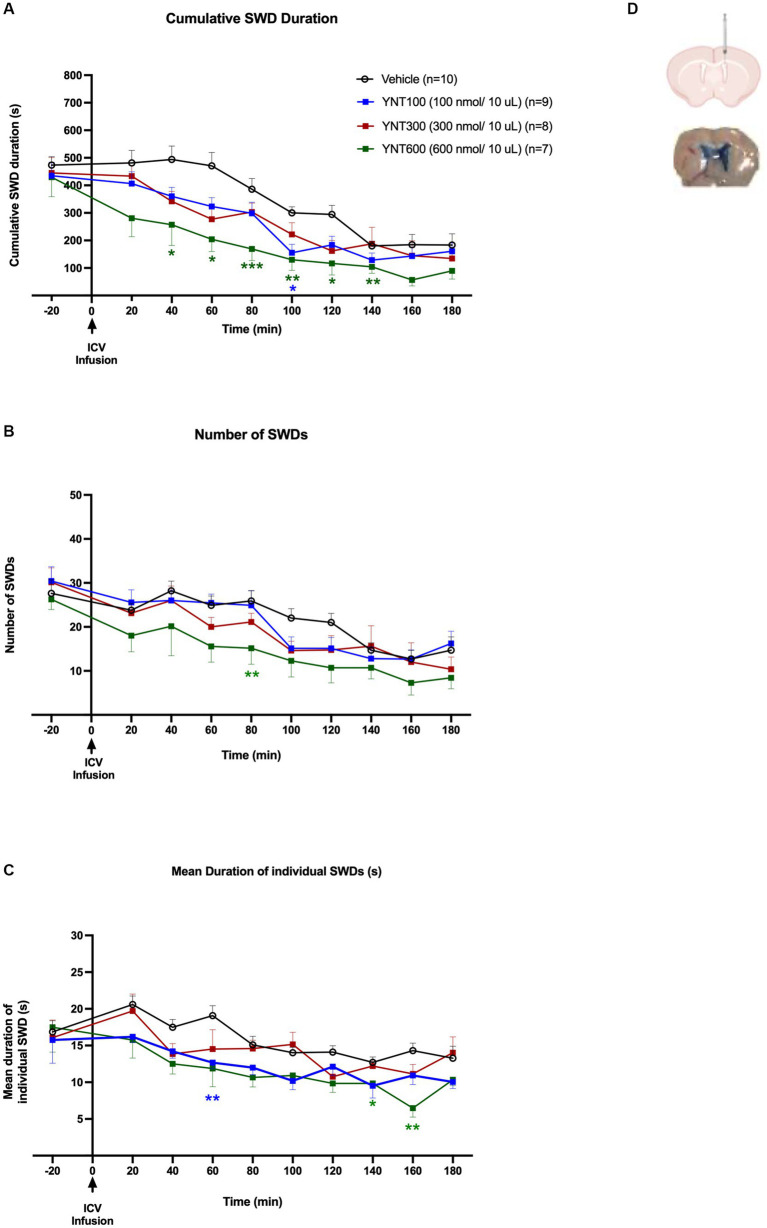
Effect of the ICV administration of YNT-185 on SWDs. The impact of ICV injection of 100 nmol/10 μL (*n* = 9), 300 nmol/10 μL (*n* = 8), or 600 nmol/10 μL (*n* = 7) of YNT-185 or saline (*n* = 11) on the cumulative seizure duration **(A)**, the number of seizures **(B)**, and the mean duration of individual seizures in adult GAERS **(C)**. 600 nmol/10 μL of YNT-185 induced a significant decrease in SWD duration, number and mean duration of starting within the 40 min post-injection period (See Supplementary Figure S2 for representative EEG traces from 600 nmol/10 μL of YNT-185 and saline injected GAERS). Data expressed as mean ± SEM. (*p < 0.05, **p < 0.01, *** *p* < 0.001). Inset figure shows the traces of methylene blue determining the sites of ICV injection **(D)**.

**Figure 2:**
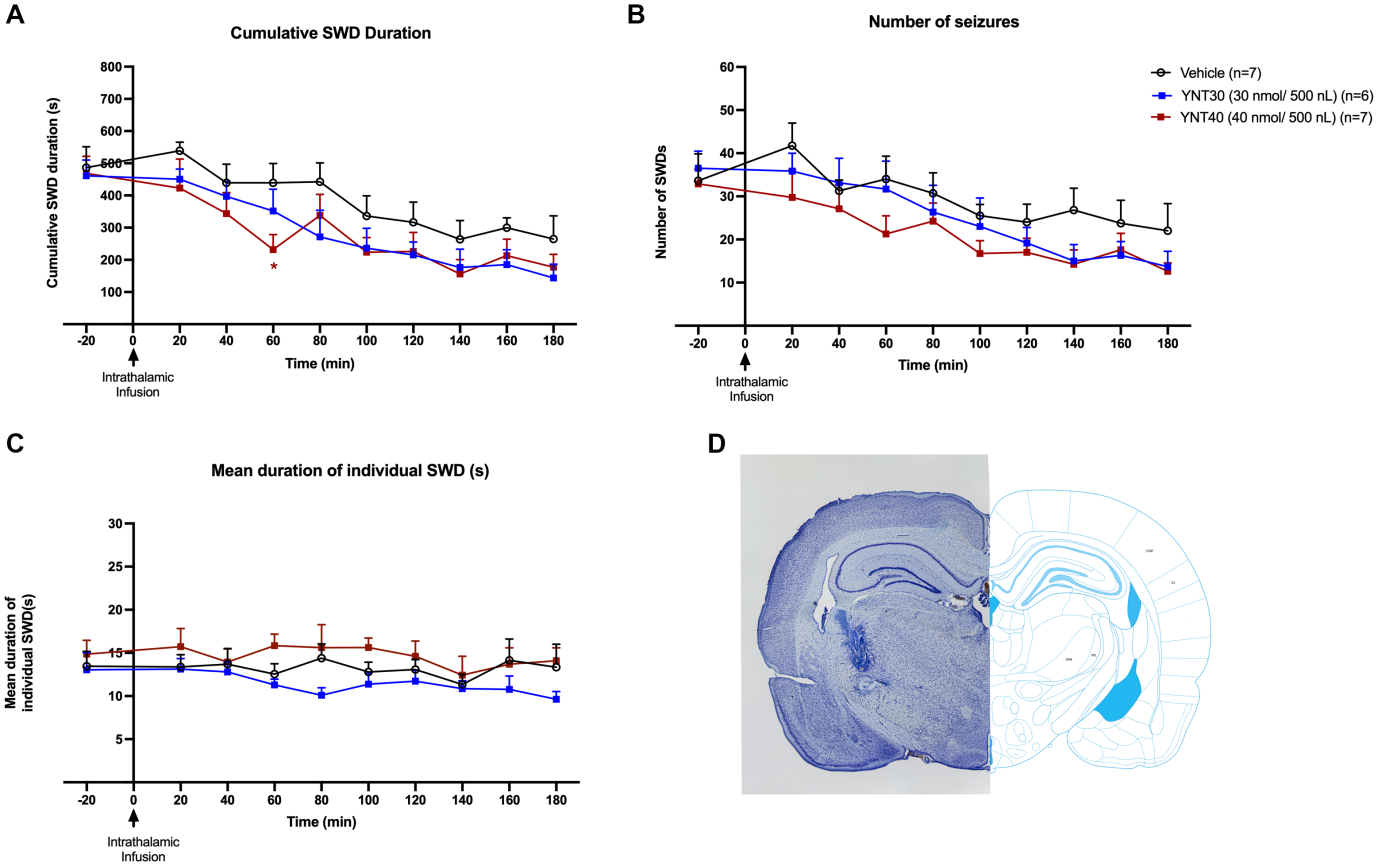
Effect of bilateral VB administration of YNT-185 on SWDs. The impact of YNT-185 injection into the bilateral VB injections of either 30 nmol/500 nL (*n* = 6), 40 nmol/500 nL (*n* = 8) of YNT-185, or 500 nL saline (0.9% NaCl solution) (*n* = 7) on the cumulative seizure duration **(A)**, the number of seizures **(B)**, and the mean duration of individual seizures **(C)**. Intrathalamic microinjections of YNT-185 did not produced any statistically significant effect on SWDs (*p* > 0.05). Data expressed as mean ± SEM. Inset figure shows the traces of bilateral guide cannula traces placed over the VB on thionine stained coronal sections **(D)**.

**Figure 3:**
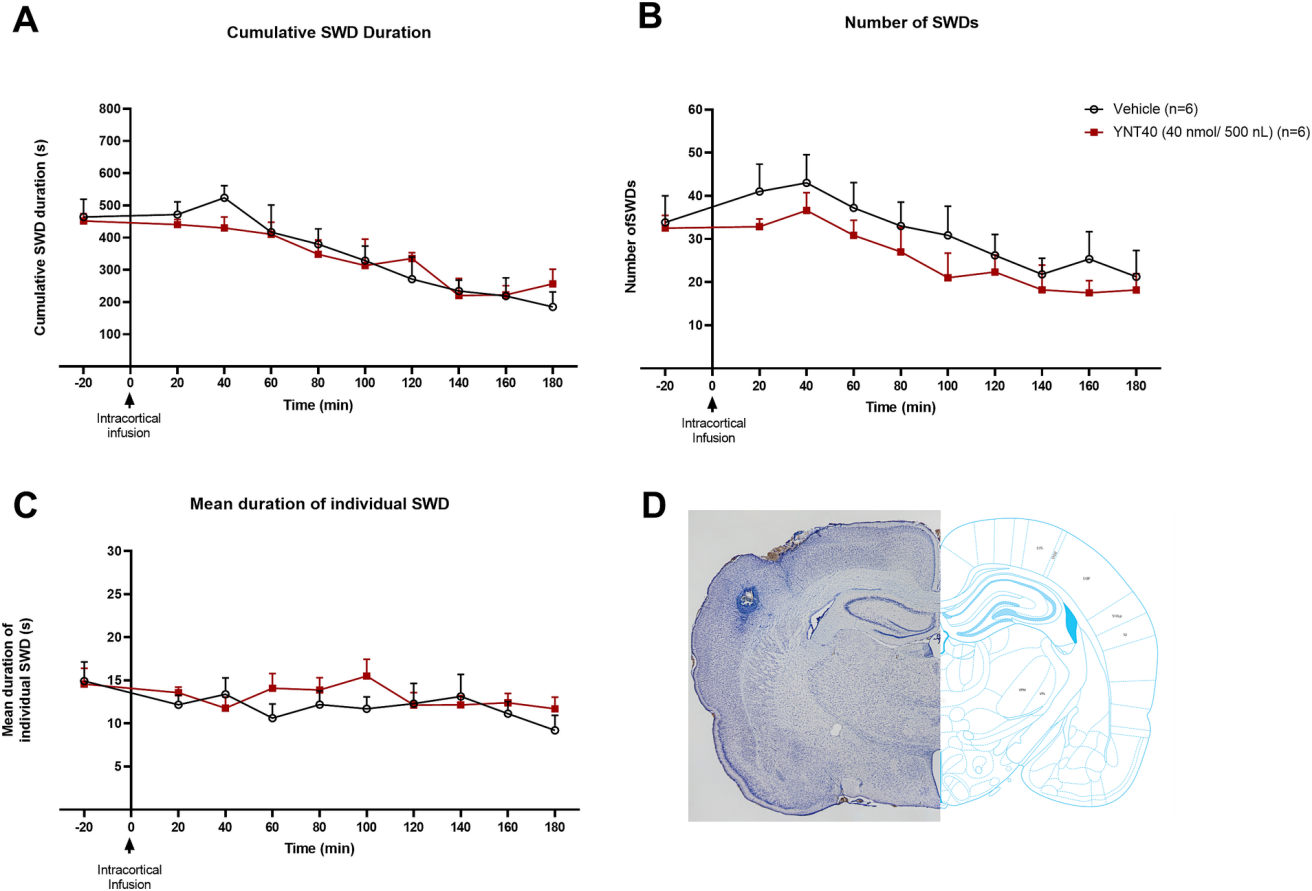
Effect of bilateral S1 administration of YNT-185 on SWDs. The impact of YNT-185 injection into the bilateral S1 injections of 40 nmol/500 nL (*n* = 6) of YNT-185 or 500 nL saline (0.9% NaCl solution) (*n* = 6) on the cumulative seizure duration **(A)**, the number of seizures **(B)**, and the mean duration of individual seizures **(C)**. There was no statistically significant effect of YNT-185 injected bilaterally into the S1 on SWDs compared to vehicle (*p* > 0.05). Data expressed as mean ± SEM. Inset figure shows the traces of bilateral guide cannula traces placed over the S1 on thionine stained coronal sections **(D)**.

For the VB or S1 groups, after the recordings, the rats were decapitated under anesthesia, and their brains were isolated and placed in a 30% formalin-sucrose mixture. Frozen sections were cut at 40 μm on a cryostat (Thermo Fisher, Cryotome™ FSE Cryostats, FE Model, 230 V 50 Hz) and stained with thionin for light microscopic examination of injection sites according to Paxinos and Watson rat brain atlas (Figures 1D, 2D and 3D). Only animals with correctly placed cannulas were included in this study.

### Western blot

The cortex and thalamus tissues were dissected and then immediately snap-freezed using liquid nitrogen. The dissected tissues were stored at −80°C until protein extraction. The tissues were homogenized in the ice-cold RIPA lysis buffer (50 mM Tris–HCl, pH 7.4, 150 mM NaCl, 1% NP-40, 0.25% sodium deoxycholate, 1 mM EDTA, 1 mM PMSF, 1 mM sodium orthovanadate, 1 mM sodium fluoride with 1X protease inhibitor cocktail) using a tissue Dounce homogenizer. The homogenized samples were then sonicated by Omni Ruptor 4,000 sonicator approximately for 1 s. The lysates were centrifuged at 14.000Xg for 15 min at 4°C to collect the supernatants. The concentration of extracted protein was quantified using Bradford protein assay (Bio-Rad, USA), and 20 μg of protein from each sample was separated by 10% SDS-polyacrylamide gel electrophoresis, then transferred to a nitrocellulose membrane using a semi-dry transfer system (40 min, 25 V) (Bio-rad, Transblot). The membranes were incubated with 5% bovine serum albumin for 1 h at RT. Afterward, the membranes were incubated with primary antibodies against OX2R (1:1000, Thermo Fisher Scientific, USA), and β-actin (1,10.000, Thermo Fisher Scientific, USA) overnight at 4°C. The membrane was rinsed three times with TBS-T for 10 min., and then probed with appropriate secondary antibodies 1:5000 (Rabbit for OX2R and mouse for β-actin) for 1 h at RT and then washed three times with TBS-T for 10 min. The protein bands were visualized by an enhanced chemiluminescence reagent (SuperSignal™ West Femto Maximum Sensitivity Substrate, Thermo Scientific, USA). Finally, the relative densities of the protein bands were analyzed by Image Lab software (Bio-Rad, USA).

### Data analysis

Statistical analysis was performed using the GraphPad Prism version 9.0.1 program. The effect of YNT-185 on SWDs: (1) The cumulative seizure duration (2) the number of seizures and (3) the duration of individual seizures were compared by two-way analysis of (Two factors: “Treatment and Time”) variance (ANOVA) using Tukey’s multiple comparison test. The relative expression of OX2R protein bands were quantified by ImageLab software. Unpaired t test was used to compare the ratio of OX2R to actin levels in the brain tissues. For statistical analysis of the percentage of SWS in the 600 nmol YNT-185 injection and control groups compared to their baseline EEG values, a repeated-measures analysis of variance (ANOVA) design with two factors, “time” and “treatment,” followed by the Bonferroni test, was used. For the comparison of spectral characteristics of the 600 nmol YNT-185 injection group, a repeated-measures ANOVA design with two factors, “treatment” and “frequency bands” was applied to the mean powers in the four frequency bands. Data were presented as mean ± S.E.M. *p < 0.05 is considered significant.

## Results

### Effect of intracerebroventricular (ICV) microinjections of YNT-185 on SWDs

In the first group of the experiments, we investigated the effect of ICV injection of 100 nmol/10 μL (*n* = 9), 300 nmol/10 μL (*n* = 8), or 600 nmol/10 μL (*n* = 7) of YNT-185 or saline (*n* = 11) on absence seizures in GAERS (Figures 1A–C). ICV injection of YNT-185 markedly reduced cumulative SWD duration in adult GAERS compared to saline injected vehicle group [*F*(3, 30) =14.07; *p* < 0.0001]. Tukey’s multiple comparison tests revealed that 600 nmol/10 μL of YNT-185 induced a significant decrease in SWD duration starting from the 40 min post-injection until the 140th min post-injection period (*p* < 0.001; Figure 1A). Representative EEG traces from 600 nmol/10 μL of YNT-185 and saline-injected GAERS clearly demonstrate the YNT-185 effect on SWDs (Supplementary Figure S2). 100 nmol/10 μL of YNT-185 induced a significant decrease in the duration of SWDs at 100 min post-injection (*p* = 0.01; Figure 1A). ICV injection of YNT-185 produced a significant effect also on SWD number [*F*(3, 30) =3.29; *p* = 0.03]. 600 nmol/10 μL of YNT-185 significantly decreased the number of SWDs at 80 min post-injection period (Figure 1B; *p* < 0.01). The mean duration of each SWD was also affected by the ICV injection of YNT-185 [*F*(3, 30) = 8.656; *p* = 0.0003]. 100 nmol/10 μL of YNT-185 significantly decreased the mean duration of SWDs at 60 min post-injection period (*p* < 0.01); this effect was observed at 140–160 min of post-injection period for 600 nmol/10 of YNT-185 injected group (Figure 1C). Interestingly, the dose of 300 nmol did not produce any statistically significant effect on cumulative SWD duration, number of SWDs, and mean duration of each SWD (Figures 1A–C). These results suggest that the inhibitory effect of YNT-185 on SWDs may not correlated with the doses of OX2R agonist YNT-185.

Thereafter, intraparenchymal doses correspond to the doses where the most pronounced effect is observed in ICV administration. So, we administered the OX2R agonist, YNT-185 intraparenchymally at the doses (either 30 or 40 nmol/500 nL) at which its effect on SWDs was most pronounced when administered ICV (600 nmol/10 μL).

### Effect of intrathalamic microinjections of YNT-185 on SWDs

In the second group, the effect of bilateral VB injection (for both complexes simultaneously) of YNT-185 on absence seizures was evaluated. GAERS received bilateral VB injections of either 30 nmol/500 nL (*n* = 6), 40 nmol/500 nL (*n* = 7) of YNT-185, or 500 nL saline (0.9% NaCl solution) (*n* = 7) (Figures 2A–C). Intrathalamic microinjections of YNT-185 slightly reduced the SWDs (both duration and number) on the EEG but this effect was not statistically different between groups (*p* > 0.05; Figures 2A–C). In the 40 nmol/500 nL YNT-185 injected group, this decrease in SWD duration was significant in the 60 min post-injection period compared to vehicle as revealed by Tukey’s multiple comparison test (*p* = 0.04; Figure 2A). The cumulative duration, number, and mean duration of SWDs did not differ across groups (Figures 2A–C). The number of SWDs on the EEG was slightly reduced in the 40 nmol/500 nL YNT-185 injected group at 60 min post-injection, but this effect was not statistically different (*p* > 0.05; Figure 2B).

### Effect of intracortical microinjections of YNT-185 on SWDs

In the third group, we examined the effect of bilateral S1 injection of YNT-185 on SWDs in GAERS. GAERS were administered bilateral S1 injections of 40 nmol/500 nL (*n* = 6) of YNT-185 or 500 nL saline (0.9% NaCl solution) (*n* = 6). There was no statistically significant effect of YNT-185 injected bilaterally into the S1 on SWDs compared to vehicle, including the cumulative SWD duration the number of SWDs, and the mean duration of SWDs on the EEG (*p* > 0.05; Figures 3A–C).

### Effect of YNT-185 on slow-wave sleep and spectral characteristics of background EEG

**Figure 4:**
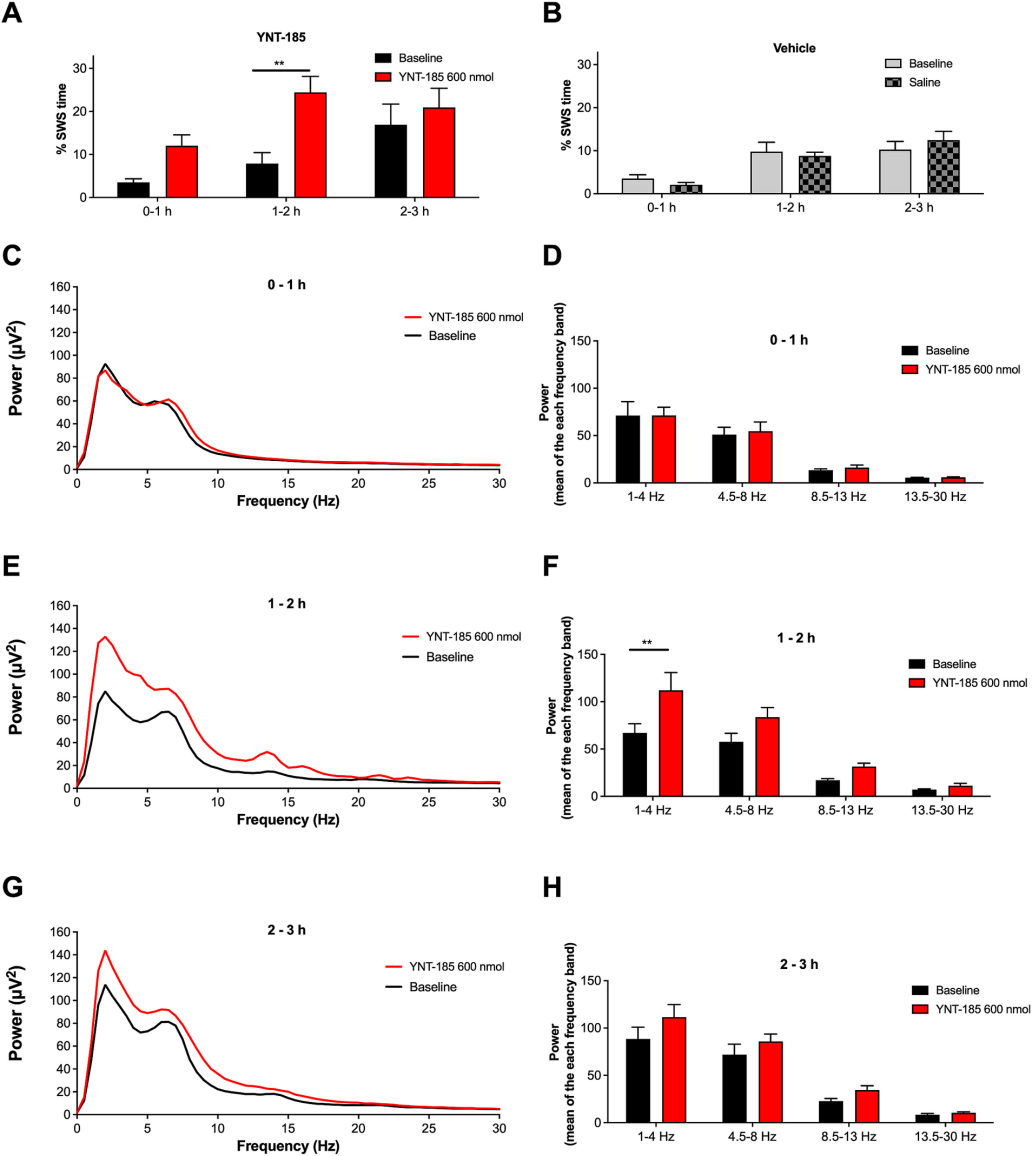
Effect of YNT-185 on percentage of slow-wave sleep and spectral characteristics of background EEG. Rats administered with 600 nmol of YNT-185 showed a significant increase in the time spent in SWS during the 1–2 h post-injection period compared to their baseline EEG recordings **(A)**. The saline injection in vehicle group did not affect the percentage of SWS when compared to that in their baseline EEG recordings **(B)**. The absolute power spectra of the EEG obtained 0-to 1 h, 1-to 2 h and 2- to 3 h post injection period in 600 nmol ICV YNT-185 group **(C,E,G)**. The mean power for delta, theta, alpha, and beta bands during 0-to 1 h, 1-to 2 h and 2- to 3 h post injection period **(D,F,H)**. Repeated measures analysis of variance test revealed significant effect of the treatment on the delta band power of the EEG frequency spectrum. Data expressed as mean ± S.E.M. **p < 0.01. 600 nmol ICV YNT-185 (*n* = 7 rats); vehicle (*n* = 10 rats).

To determine whether YNT-185 affects sleep states in GAERS, we examined the spectral characteristics of EEG recorded from the 600 nmol ICV YNT-185 group, where YNT-185 strongly suppressed the SWDs. We scored slow-wave sleep (SWS), characterized by the occurrence of high amplitude delta waves in the EEG. The percentage of SWS during the post-injection period in the YNT-185 group was higher than those in baseline EEG recordings [Treatment effect: *F*(1, 12) = 8.509, *p* = 0.0129; Figure 4A]. The post-hoc Bonferroni test showed that this difference was due to a higher percentage of SWS during the 1-2 h post-injection periods (*p* < 0.01). The saline injection administered to the vehicle group did not result in any alterations in the percentage of SWS when compared to that observed in its baseline EEG recordings [Treatment effect: *F*(1, 16) = 0.002, *p* = 0.96; Figure 4B]. The spectral characteristics of EEG recorded from the 600 nmol ICV YNT-185 group, in which YNT-185 strongly suppresses SWDs, showed a significant treatment effect on the EEG band powers [*F*(1, 12) = 5.135; *p* = 0.0427] during the 1–2 h post-injection period (Figures 4E,F). Post-hoc Bonferroni test revealed that this difference was due to higher power in the delta band following YNT-185 injection (*p* < 0.01). No difference was observed in terms of band powers during 0–1 h or 2–3 post-injection periods [Treatment effect: *F*(1, 12) = 0.0505, *p* = 0.8260; *F*(1, 12) = 1.928, *p* = 0.1902, respectively; Figures 4C,D,G,H].

### OX2R protein levels in the cortex and thalamus

**Figure 5:**
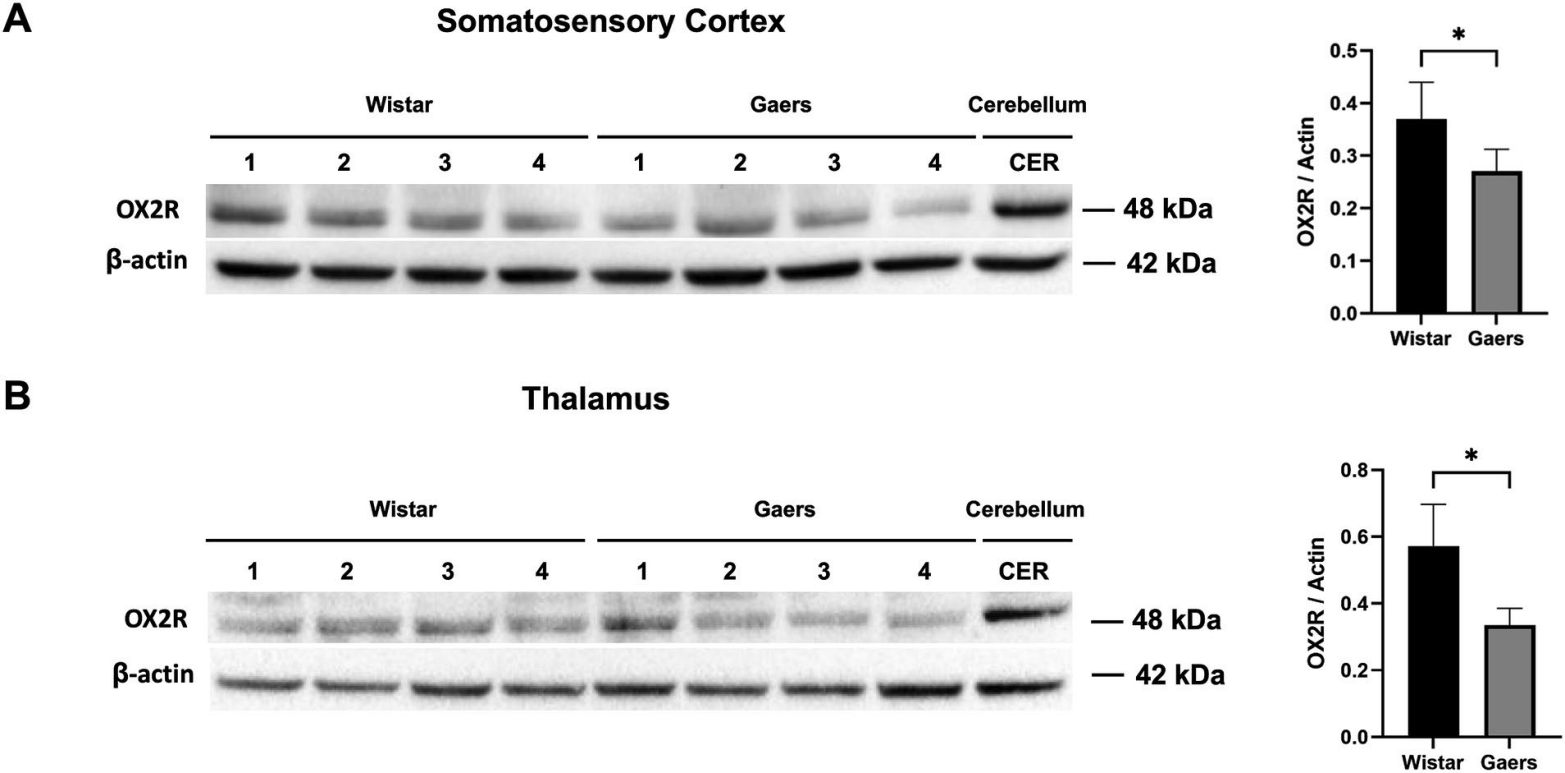
Western blot analysis of OX2R protein levels. Western blot analysis of OX2R expression in somatosensory cortex **(A)** and thalamus **(B)** of adult naïve GAERS and Wistar rats. Relative expression of OX2R protein bands in were quantified by ImageLab software. Bar graphs on the right show that OX2R protein levels in GAERS (*n* = 4) was significantly lower than Wistar rats (*n* = 4) in cortex and thalamus. Actin was used for internal loading control. The ratio of OX2R to actin levels was represented as bar graphs. Data expressed as mean ± S.E.M. (*p < 0.05, unpaired *t* test).

The cortex and thalamus tissues of male adults (3 months old) and age-matched Wistar rats were dissected and then the protein levels of OX2R were evaluated by western blot analysis in the cortex and thalamus of GAERS and Wistar rats. According to human protein atlas data, the cerebellum has the highest OX2R level.^1^ Therefore we used the cerebellum as positive control (Figures 5A,B). We demonstrated that the OX2R protein levels in GAERS were significantly lower than in Wistar rats in the cortex (*p* = 0.048; Figure 5A) and thalamus (*p* = 0.012; Figure 5B).

## Discussion

The present study demonstrates that ICV microinjection OX2R agonist YNT-185 significantly reduces the incidence of SWDs in GAERS. In contrast, intrathalamic and intracortical administration of YNT-185 did not produce a significant effect on SWDs, whereas the dose range for parenchymal injection has been selected in accordance with the ICV administration. Further, the highest dose of YNT-185 administrated into the lateral ventricle resulted in a significant increase in the percentage of SWS and 1–4 Hz delta frequency band on the EEG during the 1–2 h post-injection period in which YNT-185 significantly suppressed absence seizures (SWDs). These findings imply that the effect of YNT-185 on SWDs could be related to its modulation of wake/sleep states. In addition, OX2R expressions in the cortex and thalamus of adult GAERS were significantly lower with respect to Wistar rats. The results suggest that OX2R signaling can be related to the occurrence of SWDs and might play a role in the pathophysiology of absence epilepsy.

There is limited research on the involvement of orexinergic signaling in genetic generalized epilepsies. OX1R protein levels were reduced in the thalamus and somatosensory cortex of adult WAG/Rij rats compared to non-epileptic controls, whereas these differences were not seen in 25 days-old WAG/Rij rats those SWDs were not observed yet (15). These findings lead to the hypothesis that the age-dependent development of SWDs was associated with a down-regulation of orexin protein level in the thalamocortical network and orexin agonists can reduce the incidence of SWDs. Supporting that, our results confirmed that ICV microinjection of OX2R agonist YNT-185 reduced the incidence of SWDs in adult GAERS with reduced OX2R protein levels in the cortex and thalamus. In all, it seems that the thalamocortical circuit underlying the generation of SWDs is characterized by a dysregulation of orexins and/or orexin receptors in genetic absence epilepsy rat models and contributes to the pathogenesis of absence epilepsy. As with OX1R in WAG/Rij rats, it is important to investigate whether or not OX2R expression in GAERS is age-dependent (15). Therefore, to gain a better understanding of the relationship between orexinergic signaling and the development of SWD, age-related changes in OX2R expression in GAERS need to be demonstrated.

Research from animal models of epilepsy indicates that orexins show a proconvulsive effect and are deleterious to the pathophysiology of epilepsy. As a result, blocking orexin receptors and/or downregulation of orexin levels can attenuate seizure activity in various epilepsy models (9, 10, 20, 21). However, a few studies on animals have indicated the opposite: orexins were found to reduce epileptic activity *in-vitro* (22) and have a beneficial effect in reducing the learning and memory impairments in PTZ-kindled epilepsy rats (23). As presented here, orexin agonists might have also a beneficial effect in alleviating absence seizures. Consequently, the goal of future research should be to identify the causes of these discrepancies in orexin effects in different epilepsy models.

### Absence seizures, sleep, and orexin signaling

The main function of orexinergic signaling is to regulate sleep–wake states (24) and its deficiency is associated with narcolepsy (25, 26). Consistently, OX2R agonist YNT-185 ameliorates narcolepsy symptoms and markedly increases wakefulness time in mice in a mouse model of narcolepsy (27). In mice, activation of orexin neurons causes a significant increase in wakefulness and a decrease in sleep time, including both REM and NREM sleep (28). The relationship between sleep and absence epilepsy has been widely investigated. It has been shown that a well-documented relationship exists between SWDs and the vigilance level (29, 30). When emerging from wakefulness, absence seizures usually occur during the transition between wakefulness and sleep, less often during high arousal states such as physical and engaging mental activity (31). SWDs are absent during deep slow-wave sleep and REM sleep in both people with genetic generalized epilepsy and in the genetic models (32, 33). Supporting this evidence, our results indicated a significant increase in the percentage of SWS and 1–4 Hz delta frequency band on the EEG. These findings imply that the anti-absence effect of YNT-185 observed in GAERS might be related to its regulatory effect on sleep–wake states. It is also important to note here that, in rodent models of genetic absence epilepsy there is a natural course of SWDs being a relatively higher number at the beginning of the light phase of the 12:12 light–dark and decreases over time (31). This phenomenon was also noted here, where a tendency to decrease SWDs over time was seen in all experimental groups including vehicle. In further studies, the possible reasons for this pattern and the effect of orexinergic ligands on sleep–wake states in genetic generalized epilepsies need to be clarified.

In contrast to ICV results, intrathalamic and intracortical microinjections of YNT-185 did not produce such a significant effect on SWDs. The dose of 600 nmol of ICV YNT-185 on SWDs in GAERS produced the most pronounced effect whereas the lower doses of 100 and 300 nmol did not lead to any significant effect on SWDs, suggesting that the inhibitory effect of YNT-185 on SWDs may not be positively correlated with its concentration. A better elucidation of YNT-185’s impact on target brain regions especially to comprehend the YNT-185 concentrations in cerebrospinal fluid, receptor binding profile, and efficacy is needed in further *in vitro* studies. Moreover, the involvement of other brain regions in which the OX2Rs are highly expressed such as the hypothalamus needs to be explored in terms of YNT-185 efficacy on SWDs.

Limited studies have reported the excitatory actions of orexins on non-specific thalamic nuclei namely some intralaminar and midline thalamic nuclei but do not alter the membrane properties of neurons in primary sensory (“first-order”) or higher-order thalamic nuclei including VB (34, 35). Excitatory action of orexins is likely mediated via direct OX2R activation that has a greater expression in the thalamus compared to OX1R or indirectly by activating wake-promoting pathways projecting to the thalamus (34–36). In this study, the microinjections were administrated into the VB complex of the thalamus, which may be the reason that no significant effect on SWDs was observed. Another reason could be the reduced OX2R expression observed in the cortex and thalamus of adult GAERS. On the other hand, the intralaminar nuclei which are likely mediated via OX2R activation have also been implicated to play a role in the SWDs in animal models of absence epilepsy (37, 38). Thus, it appears that neuronal activity within the thalamocortical circuitry is differentially excited by orexins. Further pharmacological experiments on genetic models investigating the modulatory role of orexin in different non-specific thalamic nuclei may elucidate the structures underlying the absence seizure modulatory effect of orexin signaling.

The somatosensory cortex (S1) extends widely from anterior to posterior. It is most probable that the intracortical injection in our study only targets one place per hemisphere, or a small percentage of the entire S1 as observed in Figure 3. For that reason, YNT’s impact on SWDs might be lessened. As a result, the observations in our study however cannot rule out the idea that OX2R in S1 regulates absence seizures. Deep cortical layers of S1 critically involved in the generation and maintenance of SWDs have been found to be sensitive to orexin (34, 39) and selectively projecting to the thalamus. In order to better understand the function of orexinergic activity in S1, it is important to explore the possibility of using pharmacological and genetic manipulations of deep cortical layers to modify the absence phenotype. These manipulations should be tested in absence epilepsy models.

### Interaction of hypothalamic orexin signaling and thalamocortical circuitry

We still need to understand whether this modulatory effect of orexin signaling mediation is on the cortical or subcortical level. Since orexin neurons are localized in the lateral hypothalamus and have extensive intracortical and thalamic projections in the rodent brain, the possible interaction of hypothalamic orexin signaling and thalamocortical circuitry involving essentially the cortex and thalamus that correlate with spindles, delta waves, and SWDs (40) needs to be addressed here. Thalamocortical cell firing is largely controlled by the thalamic reticular nucleus (TRN), which is a thin sheet of GABAergic cells exerting a strong inhibition control over thalamocortical relay cells (41). The neuronal activity of TRN cells is also directly modulated by extra-thalamic GABA inputs, and sleep–wake neuromodulators including orexin (41, 42). GABAergic neurons in the lateral hypothalamus (LH), a brain area consisting of neurochemically heterogeneous neuronal populations implicated in the modulation of arousal, selectively inhibit TRN neurons and promote wakefulness (43). The LH also receives prominent cortical projections from the medial prefrontal and orbitofrontal cortices (44, 45). SWDs have been recorded in the lateral hypothalamus of Wistar rats (46) and the hypothalamic activation during stage 2 kindling in the genetic absence epilepsy rat model (GAERS) was significantly higher compared to non-epileptic control rats (47). Supporting these findings, single unit recordings from the LH during spontaneous SWDs in freely moving GAERS rats showed that the neuronal activity in the LH is decreased and correlated during SWDs (48). These results clearly implicate that the hypothalamic signaling and thalamocortical circuitry interact and modulate each other on the level of arousal and absence seizures.

## Conclusion

Present results revealing a suppressive effect of OX2R agonist on SWDs and an increase in sleep activity together with a significantly reduced expression of OXR2 in the thalamus and cortex in GAERS provide direct evidence about the involvement of orexin signaling as a potential player in the pathophysiology of absence epilepsy. However, the inhibitory effect of YNT-185 on SWDs seems not to be correlated with the doses of YNT-185. The findings contribute to a better knowledge of the effectiveness of orexin neuropeptides in GAERS absence seizures, which could be targeted by therapeutic intervention for absence epilepsy.

## Data availability statement

The raw data supporting the conclusions of this article will be made available by the authors, without undue reservation.

## Ethics statement

The experiments were performed in Acibadem Mehmet Ali Aydinlar University, Laboratory Animal Application and Research Center (ACU-DEHAM, HDK-2022/36). The study was conducted in accordance with the local legislation and institutional requirements.

## Author contributions

AT: Writing – review & editing, Data curation, Methodology, Software. NM: Data curation, Methodology, Writing – review & editing. EE: Data curation, Methodology, Writing – review & editing. ÖS: Data curation, Methodology, Writing – review & editing. MÇ: Data curation, Methodology, Writing – review & editing. DÖ-A: Data curation, Methodology, Writing – review & editing. ÖA: Data curation, Methodology, Writing – review & editing. ZM: Writing – review & editing, Conceptualization. NÇ: Conceptualization, Formal analysis, Funding acquisition, Investigation, Project administration, Supervision, Writing – original draft, Writing – review & editing. FO: Conceptualization, Funding acquisition, Investigation, Project administration, Supervision, Writing – original draft, Writing – review & editing, Resources, Visualization.

## Footnotes

^1^ https://www.proteinatlas.org/ENSG00000137252-HCRTR2/tissue
