# Supplementary material for: Involvement of orexin type-2 receptors in genetic absence epilepsy rats
Source: Front Neurol. 2023 Nov 30;14:1282494. doi: 10.3389/fneur.2023.1282494 (PMC10721972; doi:10.3389/fneur.2023.1282494)
Supplement: Supplementary file 1 [file Data_Sheet_1.docx]

**Supplementary Information**

**Involvement of orexin type-2 receptors in genetic absence epilepsy rats**

Toplu A.^1, 2^, Mutlu N.^3^, Erdeve E. T. ^4^, Sariyildiz O. ^2^, Çelik M.^5^, Öz Arslan D.^2,5,6^, Akman Ö.^7^, Molnár Z.^8^, Çarçak N.^2,9^, Onat F.^2, 10^

**Corresponding author:**

Prof. Dr. Filiz Yılmaz Onat

Medical Pharmacology

School of Medicine/ Acibadem Mehmet Ali Aydinlar University

e-mail: filiz.onat@acibadem.edu.tr

Tel: +90 (216) 500 42 53

**Short Title:** Effect of YNT-185 in GAERS

**Keywords:** Absence epilepsy, orexin 2 receptor, spike-and-wave discharge, YNT-185, epilepsy rat model

**Supplementary Figures**

**
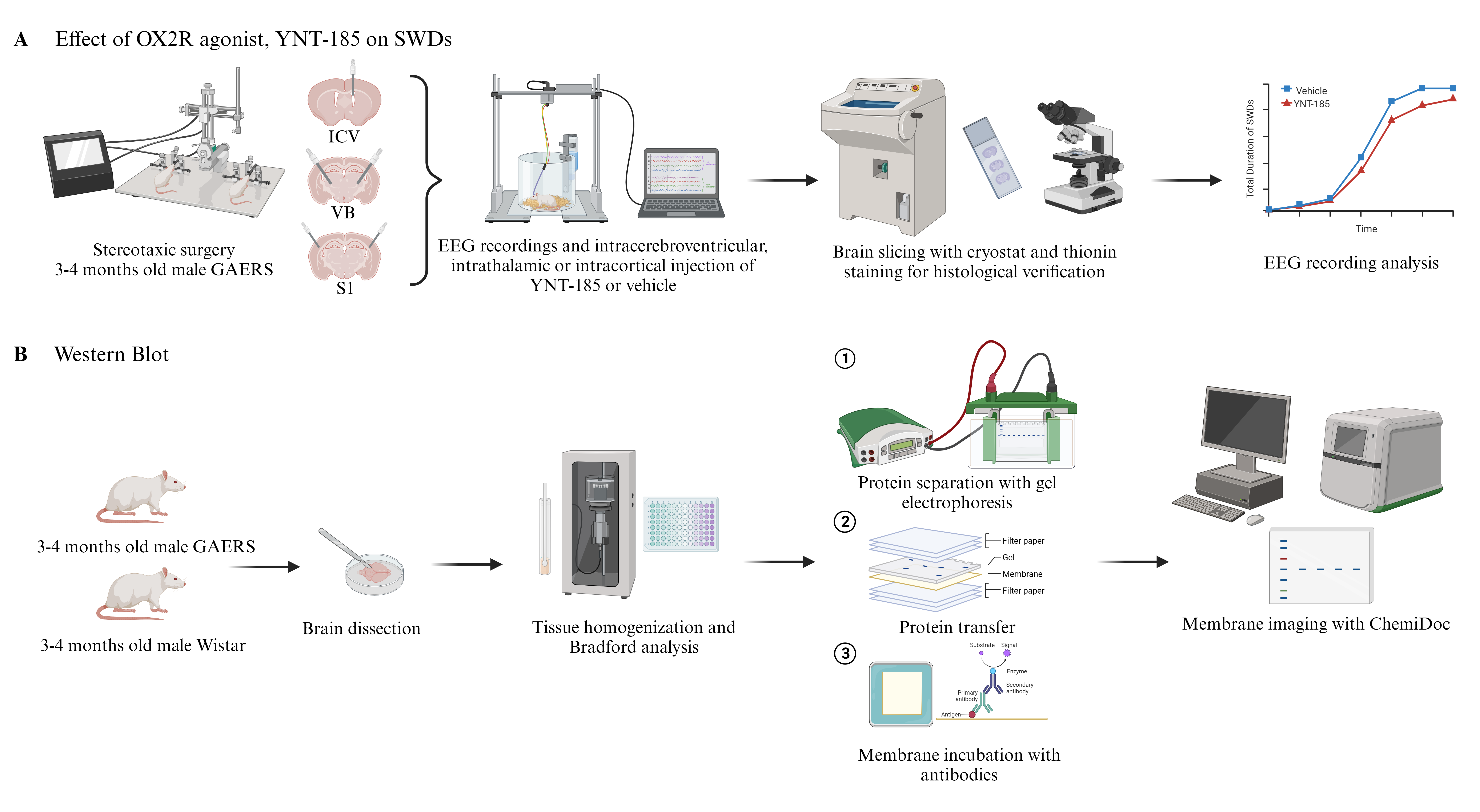
**

**Supplemantary Figure 1: Experimental design.** The effect of intracerebroventricular (ICV) (100, 300 and 600 nmol/10 µL), intrathalamic (VB; 30 and 40 nmol/500 nL) and intracortical (S1; 40 nmol/500 nL) microinjections of YNT-185 on the duration and number of spontaneous SWDs were evaluated in adult GAERS. Only animals with correct placement were included in the study **(A).** The level of OX2R expression in somatosensory cortex and projecting thalamic nuclei of adult GAERS were examined by Western blot and compared with the non-epileptic Wistar rats **(B).**


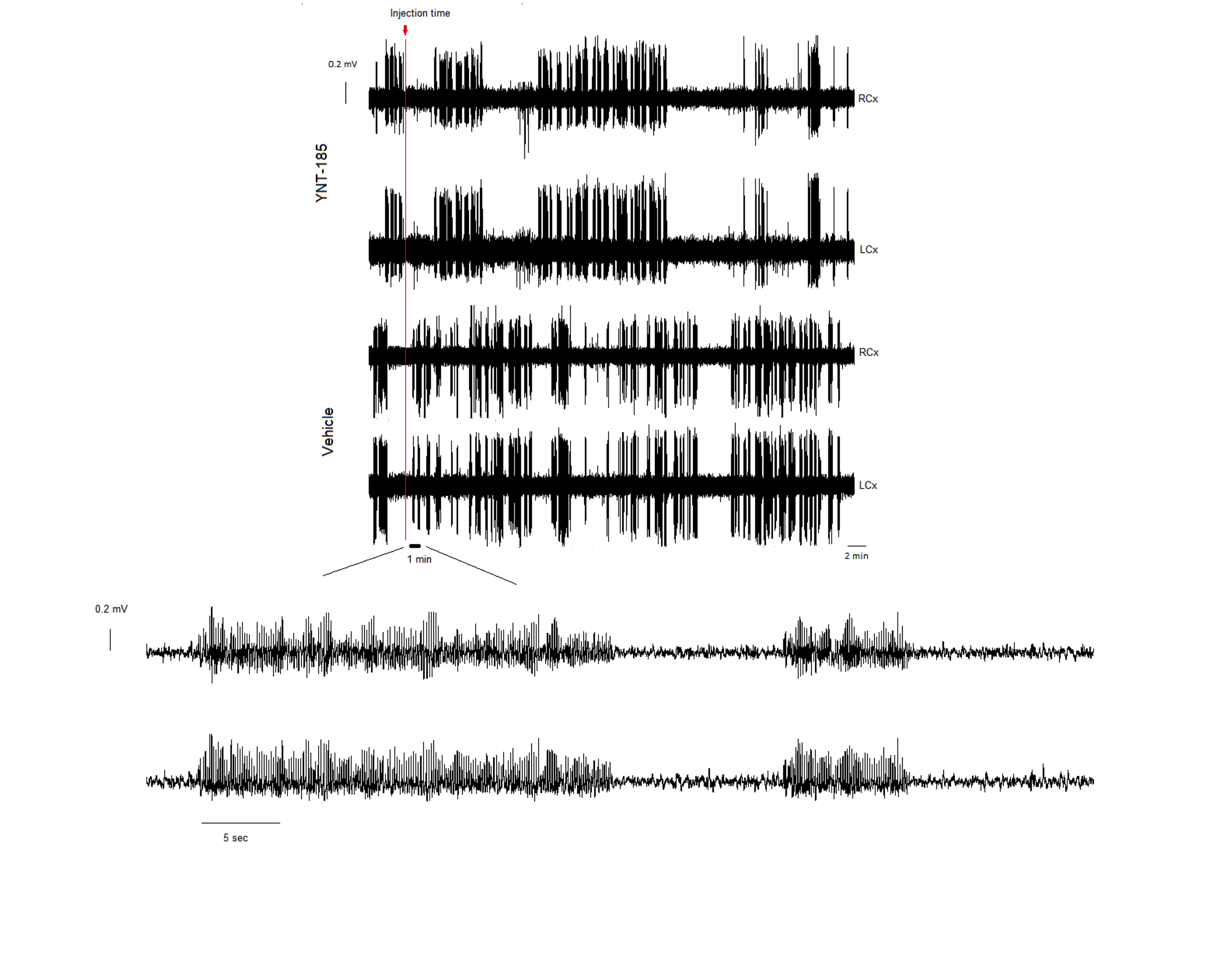


**Supplemantary Figure 2: Sample of EEG traces.** Representative 40 min EEG traces of freely moving GAERS following ICV injection of 600nmol/10 ul YNT-185 (upper two traces; from Right (R) and Left (L) side of cortex (Cx); YNT-185) and saline (lower two traces, Vehicle). Red line shows the time of ICV injections. At the bottom, a 1 min EEG traces of vehicle group was expanded in order to demonstrate the quality of EEG signals and SWD classification. SWD complexes with a train of SWD (7–11 Hz) and an amplitude at least twice that of the background EEG were found at periods longer than 1 second can be observed at the bottom expanded EEG traces.
